# Analysis of risk factors for sepsis-related liver injury and construction of a prediction model

**DOI:** 10.3389/fpubh.2024.1475292

**Published:** 2024-12-06

**Authors:** Yong He, Chi Wang, Wan He, He Zhang, Fei Ding, Ying Liu, He He, Binwu Ying, Xin Nie

**Affiliations:** ^1^Department of Laboratory Medicine, West China Hospital, Sichuan University, Chengdu, Sichuan, China; ^2^Sichuan Clinical Research Center for Laboratory Medicine, Chengdu, Sichuan, China; ^3^Clinical Laboratory Medicine Research Center of West China Hospital, Chengdu, Sichuan, China; ^4^Department of Laboratory Medicine, West China Xiamen Hospital, Sichuan University, Xiamen, Fujian, China

**Keywords:** sepsis, liver injury, early prediction, risk factors, ROC curve

## Abstract

**Background:**

Sepsis is a leading cause of mortality in critically ill patients, and the liver is a key organ affected by sepsis. Sepsis-related liver injury (SRLI) is an independent risk factor for multiple organ dysfunction syndrome (MODS) and mortality. However, there is no clear diagnostic standard for SRLI, making early detection and intervention challenging.

**Objective:**

This study aimed to investigate the predictive value of serum indices for the occurrence of SRLI in adults to guide clinical practice.

**Methods:**

In this study, we investigated the predictive value of serum indices for SRLI in adults. We retrospectively analyzed data from 1,573 sepsis patients admitted to West China Hospital, Sichuan University, from January 2015 to December 2019. Patients were divided into those with and without liver injury. Stepwise logistic regression identified independent risk factors for SRLI, and a predictive model was constructed. The model’s diagnostic efficacy was assessed using receiver operating characteristic (ROC) curve analysis.

**Results:**

Our results showed that alanine aminotransferase (ALT), gamma-glutamyl transpeptidase (GGT), carbon dioxide combining power (CO_2_-CP), antithrombin III (AT III), fibrin/fibrinogen degradation products (FDP), and red blood cell distribution width (RDW-CV) were independent predictors of SRLI. The area under the curve (AUC) of the predictive model was 0.890, with a sensitivity of 80.0% and a specificity of 82.91%, indicating excellent diagnostic value.

**Conclusion:**

In conclusion, this study developed a highly accurate predictive model for SRLI using clinically accessible serum indicators, which could aid in early detection and intervention, potentially reducing mortality rates.

## 1 Introduction

Sepsis is a severe, fatal organic dysfunction caused by a disordered body response to infection (1). Notwithstanding the current advancements in therapeutic interventions, sepsis continues to exert a substantial influence on the mortality rates of critically ill individuals (2). The liver is the largest detoxifying organ in the human body; this organ participates in the clearance of inflammatory factors or inflammatory products and plays an important role in regulating metabolic disorders and maintaining the internal environment of the body (3). Moreover, its prognostic value for patient mortality surpasses that of conventional indicators such as circulatory, renal, and central nervous system dysfunction (4). Sepsis-related liver injury (SRLI) is considered to be an independent risk factor for multiple organ dysfunction syndrome (MODS) and mortality (5). However, there is no guideline or consensus on the diagnosis of SRLI, and liver insufficiency is closely related to the mortality of septic patients; therefore, early identification and treatment of liver insufficiency are crucial.

The pathogenesis of liver dysfunction in sepsis is complex. These studies have focused mainly on the exaggerated inflammatory response, aberrant metabolism of liver microcirculation, compromised mitochondrial function of hepatic cells, dysbiosis of intestinal microbiota/endotoxins, oxidative stress and lipid peroxidation, the involvement of polymorphonuclear neutrophils (PMNs), and the activation of platelet activation factor (PAF) (6–9). The main clinical manifestations of SRLI have three aspects: cholestasis, hypoxic hepatitis, blood coagulation dysfunction, and abnormal liver function have a great, which strongly impact the prognosis of septic patients (10). Early detection and implementation of interventions are of great help to the prognosis of septic patients, and further understanding of the pathogenic mechanism of liver injury in septic patients is of great help to the treatment of patients and can effectively reduce the mortality rate of septic patients. Because the pathophysiology of SRLI has not been fully elucidated, the conventional diagnostic criteria for SRLI continue to rely on total bilirubin (TBIL) and international normalized ratio (INR) levels (11). Nevertheless, it is important to note that elevated serum TBIL and INR levels, while capable of diagnosing SRLI, are not sufficiently sensitive indicators of liver injury and fail to promptly and accurately reflect the presence of sepsis. Other laboratory indicators, such as alkaline phosphatase (ALP), C-reactive protein (CRP), albumin (ALB) and lactate (LAC), are risk factors for SRLI in patients with sepsis (12, 13). However, as a result of the presence of numerous confounding variables, laboratory indicators fail to provide an accurate prediction of the incidence of SRLI. Hence, it is imperative to screen out clinical or laboratory indicators to establish sensitive, precise, and convenient predictive indicators for timely detection of SRLI, thereby playing a crucial role in mitigating mortality rates among patients with SRLI.

## 2 Methods

### 2.1 Source of data and study population

We conducted a retrospective single-center study using data from the West China Hospital of Sichuan University. The study included all patients diagnosed with sepsis and septic shock between January 2015 and December 2019. The inclusion criteria were as follows: (1) met the Sepsis-3.0 diagnostic criteria and had a sequential organ failure assessment (SOFA) score ≥ 2 (14); (2) were aged 18 years; (3) had a stay in the hospital ≥7 days; and (4) Approval from the hospital’s ethical committee; (5) complete clinical data. The exclusion criteria for patients were as follows: (1) had a previous history of chronic liver function impairment, such as chronic hepatitis, cirrhosis, liver cancer, or fatty liver; (2) incomplete or missing clinical medical records; (3) incomplete clinical data; (4) were <18 years of age; and (5) had liver injury from non-septic causes (such as drugs, poisons or trauma). A total of 1,573 septic patients were included in this study and were classified into the SRLI group or sepsis non-liver injury group according to whether liver injury occurred within 28 days after admission. The diagnostic criterion for SRLI was a bilirubin level ≥ 34.2 μmol/L (2 mg/dL) coupled with coagulation abnormalities characterized by an international normalized ratio (INR) > 1.5 (4, 15).

### 2.2 Data collection

Basic data, including age and sex, were collected from eligible patients. Clinical data and results were obtained from Hospital Information System (HIS) and Laboratory Information Management System (LIS). The first clinical serum test results after hospitalization were collected, encompassing 58 indicators related to biochemical, inflammatory, immune, blood cell, and coagulation functions. These included: white blood cell (WBC) count, absolute lymphocyte (LYMPH) count, absolute neutrophil (NEUT) count, C-reactive protein (CRP) level, procalcitonin (PCT) level, alanine aminotransferase (ALT) level etc.

### 2.3 Statistical analysis

Statistical analysis was performed using SPSS 26.0 software. The mean ± SD is used to present measurement data of continuous variables, while frequencies with percentages are employed for categorical variables. Normally distributed continuous variables data are compared by *t*-test and comparisons between categorical variables were made using the chi-square test. Non-normally distributed continuous variables data are expressed as median and interquartile range (IQR) and compared using the Mann–Whitney *U* test.

#### 2.3.1 Univariate analysis

Univariate logistic regression analysis was conducted to identify potential risk factors for SRLI. Variables with a *p*-value <0.05 in the univariate analysis were selected for further multivariate analysis.

#### 2.3.2 Multivariate analysis

Stepwise logistic regression analysis was performed to identify independent risk factors for SRLI. The backward elimination method was used, where variables with a *p*-value >0.05 were removed from the model in a stepwise manner. The final model included only variables that were statistically significant (*p* < 0.05). The odds ratios (ORs) and 95% confidence intervals (CIs) for each variable were calculated to quantify the strength of association.

#### 2.3.3 Construction of the prediction model

The significant variables identified in the multivariate analysis were used to construct a predictive model for SRLI. The model was evaluated using ROC curve analysis. The AUC was calculated to assess the model’s discriminatory power. Sensitivity, specificity, and the optimal cutoff value were determined to maximize the model’s predictive accuracy.

#### 2.3.4 Additional predictive indices

To further validate the model, we also evaluated the predictive value of the ratio of ALT to CO_2_-CP and the APRI. The AUC, sensitivity, and specificity for these indices were calculated using ROC curve analysis.

## 3 Results

### 3.1 Characteristics of the study cohorts

This cross-sectional study included a total of 1,573 patients who were diagnosed with sepsis between January 2015 and December 2019 at West China Hospital. The baseline characteristics of the septic patients, classified according to the presence or absence of liver injury, are presented in Table 1. Among 1,573 septic patients, 113 (7.18%) had SRLI, the median age was 53 years [interquartile range (IQR) = 42–66 years], and approximately 65.4% were male.

**Table 1:** Characteristics of the study cohorts.

	Total *n* = 1,573	Sepsis *n* = 1,460	SRLI *n* = 113	*P*
Age (years)	55(44–68)	55(44–68)	53(42–66)	0.227
Male, *n* (%)	907(57.7)	832(52.9)	75(65.4)	0.06
IL-6 (pg/mL)	67.57(23.81–228.43)	62.7(22.35–207.73)	162.6(69.51–1,079)	<0.01
MYO (ng/mL)	105.95(38.99–391.38)	98.46(35.82–351.23)	323(83.09–1136.47)	<0.01
PCT (ng/mL)	3.41(0.63–22.5)	2.89(0.57–20.89)	14.46(3.46–47.3)	<0.01
ALT (U/L)	31(17–69)	29(16–61)	88(36.5–285)	<0.01
AST (U/L)	41(23–87)	38(22–77)	161(51–500)	<0.01
PO_4_ (mmol/L)	0.92(0.67–1.22)	0.91(0.67–1.2)	1.02(0.65–1.61)	<0.01
CHOL (mmol/L)	2.83(2.08–3.71)	2.88(2.13–3.78)	2.06(1.55–2.97)	<0.01
LAC (mmol/L)	1.8(1.3–2.8)	1.8(1.3–2.7)	2.8(1.8–5.05)	<0.01
CK (U/L)	74(34–261)	71(33–227.75)	265(75–701.5)	<0.01
TNT (ng/L)	31.7(13.7–76.3)	31.1(13.45–74.05)	44.8(19.13–95.07)	0.007
LDH (U/L)	271(195–433)	263.5(192–413)	433(252.5–849)	<0.01
DBIL (μmol/L)	7.7(4.1–16.8)	7.1(3.9–13.6)	58.4(39.35–91.2)	<0.01
HDL (mmol/L)	0.61(0.3–1.01)	0.62(0.32–1.03)	0.38(0.16–0.74)	<0.01
CK-MB (ng/mL)	2.01(0.96–4.77)	1.92(0.93–4.27)	4.34(2–10.17)	<0.01
TP (g/L)	61.4(54.6–69.3)	62(55.23–69.8)	55.5(50.8–60.55)	<0.01
IBIL (μmol/L)	41(23–87)	5.2(3.2–8.1)	9.8(5.15–21.4)	<0.01
ALP (U/L)	97(69–158)	95(69–154)	133(80–238.5)	<0.01
LDL (mmol/L)	1.21(0.59–1.96)	1.27(0.63–2)	0.69(0.2–1.17)	<0.01
β-HBA (mmol/L)	0.19(0.09–0.49)	0.19(0.08–0.47)	0.34(0.14–0.63)	<0.01
GGT (U/L)	55(26–137)	53(25.25–129)	116(48–304.5)	<0.01
PH	7.44(7.39–7.48)	7.44(7.39–7.48)	7.42(7.36–7.48)	0.013
GLB (g/L)	29.4(25.1–34.35)	29.55(25.3–34.7)	26.3(22.25–31.7)	<0.01
CA (mmol/L)	2.04(1.91–2.18)	2.05(1.92–2.18)	1.93(1.81–2.12)	<0.01
ALB (g/L)	31.2(27.2–36.2)	31.4(27.42–36.5)	28.7(24.4–32.9)	<0.01
K (mmol/L)	3.85(3.45–4.3)	3.84(3.44–4.27)	3.99(3.64–4.56)	0.01
CO_2−_CP (mmol/L)	19.8(15.9–23.43)	20.2(16.5–23.7)	15.5(10.4–18.35)	<0.01
TBIL (μmol/L)	14.4(8.8–26.35)	13.5(8.5–22.1)	71.5(48.45–112.2)	<0.01
HBDH (U/L)	208(152–332)	203.5(150–324.75)	309(188.5–536)	<0.01
TG (mmol/L)	1.39(0.95–2.11)	1.4(0.97–2.14)	1.12(0.78–1.91)	0.006
MCH (pg)	29.9(28.5–31.4)	29.9(28.5–31.3)	30.9(28.7–33)	<0.01
MCHC (g/L)	331(320–341)	331(320–341)	337(326.5–346)	<0.01
RDW-CV (%)	14.5(13.5–16)	14.5(13.5–15.9)	15.3(14.4–17.5)	<0.01
RDW-SD (fL)	46.5(42.8–51.4)	46.3(42.8–51.1)	48.6(44–56.95)	<0.01
PLT (10^9^/L)	136(73–212)	144(81–216)	60(38–112)	<0.01
WBC (10^9^/L)	10.46(6.63–16.83)	10.4(6.58–16.49)	12.89(7.63–22.05)	0.004
NEUT (10^9^/L)	8.69(4.89–14.7)	8.51(4.84–14.3)	11.13(6.31–19.35)	0.006
FIB (g/L)	4.07(2.82–5.53)	4.17(2.93–5.62)	2.74(1.52–4.31)	<0.01
ATIII (%)	64.4(50.7–79.3)	66.05(52.7–80.6)	42.6(33.8–55.75)	<0.01
DFIB (g/L)	5.52(3.54–7.7)	5.62(3.66–7.78)	4.04(2.24–6.25)	<0.01
APTT (s)	33.6(29.1–41.43)	33.1(28.9–39.5)	52.4(39.15–66.8)	<0.01
FDP (mg/L)	13.8(6.83–27.58)	13(6.57–24.85)	33.35(17–69.05)	<0.01
INR	1.21(1.08–1.4)	1.19(1.07–1.34)	1.76(1.62–2.2)	<0.01
DD (mg/L)	5.57(2.29–10.94)	4.95(2.23–9.97)	13.42(7.02–22.03)	<0.01
TT (s)	17.7(16.6–19.4)	17.0.6(16.5–19.2)	19.45(17.42–23.53)	<0.01
PT (s)	13.9(12.4–19.4)	13.7(12.3–15.4)	18.5(20.8–25)	<0.01
APRI	0.33(0.14–1.03)	0.29(0.13–0.87)	2.29(0.76–11.08)	<0.01

IL-6, Interleukin-6; MYO, myoglobin; PCT, procalcitonin; ALT, alanine aminotransferase; AST, aspartate aminotransferase; CHOL, cholesterol; LAC, lactate; CK, creatine kinase; TNT, troponin T; LDH, lactate dehydrogenase; DBIL, direct bilirubin; HDL, high-density lipoprotein cholesterol; CK-MB, creatine Kinase-MB; TP, total protein; IBIL, indirect bilirubin; ALP, alkaline phosphatase; LDL, low-density lipoprotein cholesterol; β-HBA, beta-hydroxybutyrate; GGT, gamma-glutamyl transpeptidase; PH, pondus hydrogenii; GLB, globulin; CA, calcium; ALB, albumin; K, kalium; CO_2_-CP, carbon dioxide combining power; TBIL, total bilirubin; HBDH, hydroxybutyrate dehydrogenase; TG, triglyceride; MCH, mean cell hemoglobin; MCHC, mean cell hemoglobin concentration; RDW, red cell distribution width; PLT, platelet; WBC; white blood cell; NEUT, neutrophil; FIB, Fibrinogen; AT III, antithrombin III; DFIB, derived fibrinogen; APTT, activated partial thromboplastin time; FDP, fibrinogen degradation; INR, international normalized ratio; D-D, D-dimer; TT, thrombin time; PT, prothrombin time; APRI, AST-to-platelet ratio index.

We used the variance inflation factor (VIF) to rule out multicollinearity between models. Variables with a VIF > 10 were excluded, leaving 58 serum indices for univariate analysis. The results showed significant differences in 46 indicators, including interleukin 6 (IL-6), myoglobin (MYO), and procalcitonin (PCT). Detailed results are provided in Supplementary Tables S1, S2. For example, IL-6 levels were significantly higher in the SRLI group (162.6 pg./mL, IQR = 69.51–1,079) compared to the non-SRLI group (62.7 pg./mL, IQR = 22.35–207.73; *p* < 0.01). Similarly, MYO and PCT levels were also significantly higher in the SRLI group (MYO: 323 ng/mL, IQR = 83.09–1136.47 vs. 98.46 ng/mL, IQR = 35.82–351.23; *p* < 0.01; PCT: 14.46 ng/mL, IQR = 3.46–47.3 vs. 2.89 ng/mL, IQR = 0.57–20.89; *p* < 0.01). These findings suggest that higher levels of inflammatory markers are associated with a higher risk of SRLI (Table 1).

### 3.2 Multivariate analysis revealed the independent predictive factors of the SRLI in septic patients

Multivariate analysis was performed according to age and sex, and significant variables in the univariate analysis were included in the stepwise method. The results, shown in Table 2, identified the following independent predictors of SRLI: ALT (adjusted *OR* = 1.001; 95% *CI*: 1.001–1.002; *p* < 0.001), GGT (adjusted *OR* = 1.000; 95% *CI*: 1.001–1.002; *p* < 0.001), CO_2_-CP (adjusted *OR* = 0.855; 95% *CI*: 0.81–0.902; *p* < 0.001), RDW-CV (adjusted *OR* = 1.264; 95% *CI*: 1.143–1.396; *p* < 0.001), AT III (adjusted *OR* = 0.937; 95% *CI*: 0.92–0.954; *p* < 0.001), and FDP (adjusted *OR* = 1.021; 95% *CI*: 1.01–1.033; *p* < 0.001) levels. These findings indicate that higher levels of ALT, GGT, RDW-CV, and FDP, and lower levels of CO_2_-CP and AT III, are significant risk factors for SRLI.

**Table 2:** Multivariate analyses of clinical parameters in septic patients within the first test after hospitalization.

Variables	*β*	S.E.	Wald	*P*	OR	95% CI	
						Lower	Upper
ALT	0.001	0.000	15.311	<0.01	1.001	1.001	1.002
GGT	0.001	0.000	9.689	<0.01	1.001	1.000	1.002
CO_2_-CP	−0.127	0.027	32.805	<0.01	0.855	0.810	0.902
RDW-CV	0.234	0.051	21.072	<0.01	1.264	1.143	1.396
ATIII	−0.065	0.009	48.890	<0.01	0.937	0.920	0.954
FDP	0.021	0.006	13.586	<0.01	1.021	1.010	1.033

ALT, alanine aminotransferase; GGT, gamma-glutamyl transpeptidase; CO_2_-CP, carbon dioxide combining power; AT III, antithrombin III; FDP, fibrin/fibrinogen degradation products; RDW-CV, red blood cell distribution width (RDW)-coefficient of variation.

### 3.3 ROC analysis for variables as biomarkers for SALI

**Figure 1:**
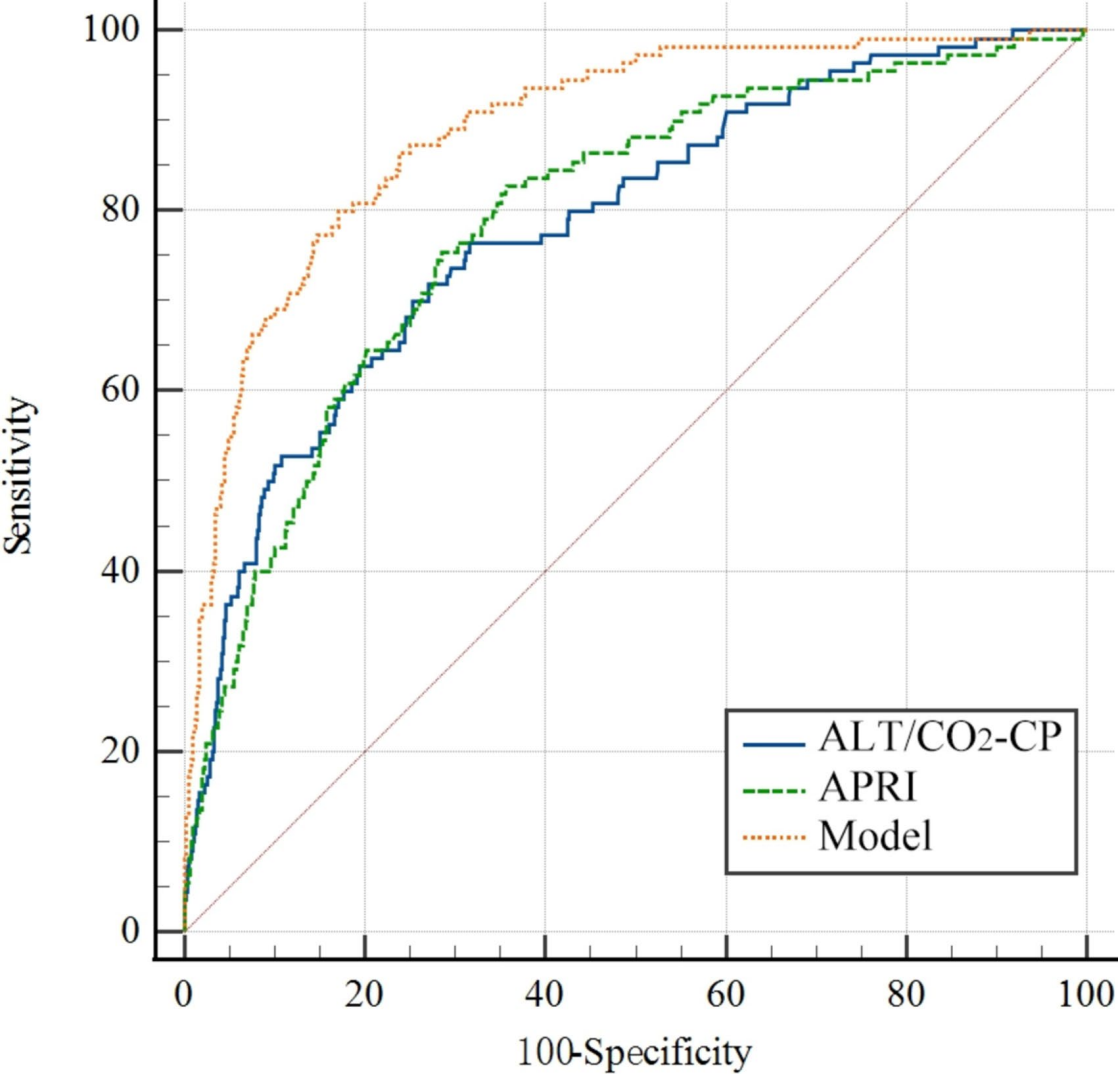
The ROC curve of the prediction model for the SRLI. The variables in the model were ALT, GGT, CO_2_-CP, RDW-CV, AT III, and FDP. APRI, AST-to-platelet ratio index; ALT, alanine aminotransferase; GGT, gamma-glutamyl transpeptidase; CO_2_-CP, carbon dioxide combining power; AT III, antithrombin III; FDP, fibrin/fibrinogen degradation products; RDW-CV, red blood cell distribution width (RDW)-coefficient of variation.

Based on the multivariate analysis, we constructed a predictive model using the following serum indices: ALT, GGT, CO_2_-CP, RDW-CV, AT III, and FDP (Table 3). The area under the ROC curve of the model was 0.890, with a sensitivity of 80.0%, specificity of 82.91%, and a cutoff value of 0.62 (Figure 1). This indicates that the model had the best diagnostic performance.

**Table 3:** The construction of prediction model for SRLI.

Variables	AUC (95% CI)	Cutoff	Sensitivity (%)	Specificity (%)	*P*
Model	0.890	0.62	80.00	82.91	<0.001
ALT/CO_2_-CP	0.791	0.46	76.99	69.17	<0.001
APRI	0.797	0.48	83.19	65.27	<0.001

APRI, AST-to-platelet ratio index; ALT, alanine aminotransferase; CO_2_-CP, carbon dioxide combining power.

Additionally, the ratio of ALT to CO_2_-CP in the model also had good predictive value, with an AUC of 0.791, a sensitivity of 76.99%, a specificity of 69.17%, and a cutoff value of 0.46 (Table 3). The AST-to-platelet ratio index (APRI) was also calculated and showed strong diagnostic efficacy for SRLI, with an AUC of 0.797, a sensitivity of 83.19%, a specificity of 65.27%, and a cutoff value of 0.48 (Figure 1).

These results suggest that the predictive model and the ALT/CO_2_-CP ratio, as well as the APRI, are valuable tools for early detection of SRLI, potentially improving patient outcomes by facilitating timely intervention.

## 4 Discussion

Sepsis remains a leading cause of mortality among critically ill patients, imposing a significant burden on global healthcare systems (16). The liver, a critical organ for detoxification and regulation of inflammatory responses, is highly susceptible to damage during the early stages of sepsis (17). This vulnerability makes the liver a prominent target organ in SRLI. Currently, there is no clear definition or uniform diagnostic standard for SRLI, underscoring the need for improved early detection methods to enhance patient survival rates. This study aimed to identify and evaluate potential risk factors for SRLI to facilitate early detection and improve patient outcomes.

Our retrospective analysis of 1,573 sepsis patients revealed an SRLI incidence of 7.18%. Previous studies have reported varying incidences of sepsis-related liver injury, ranging from 1.3 to 46.0% (18). For example, a study by Woźnica et al. (4) reported an incidence of 5.4% in a cohort of septic patients, while another study by Liang et al. (5) found an incidence of 12.5%. The discrepancies in reported incidences can be attributed to the lack of standardized diagnostic criteria and variations in patient populations. Our results (7.18%) are consistent with these studies, suggesting that the incidence of SRLI is a significant concern in sepsis management. Notably, a higher prevalence of SRLI was observed in male patients (65.4%), which aligns with prior reports (19). This gender disparity may be attributed to the protective effects of estrogen on liver function and its ability to mitigate the liver’s response to endotoxins.

In this study, our results showed that ALT, GGT, CO_2_-CP, RDW, AT III, and FDP are independent predictors of the SRLI, and a stepwise prediction model was established. We found that the AUC of the model reached 0.890, which indicates good diagnostic performance for SALI. In view of the simplicity and practicality of clinical detection, we opted to exclusively incorporate two independent indicators, ALT and CO_2_-CP_,_ into the model. Receiver operating characteristic (ROC) analysis revealed that the area under the curve (AUC) was 0.791. This approach offers the advantage of requiring patients solely to undergo biochemical serum indicator testing.

Sepsis combined with liver dysfunction is mainly characterized by hepatocellular damage, cholestasis, and regenerative dysfunction (4). ALT and GGT are well-established biomarkers of liver injury. For instance, a study by Xie et al. (13) found that elevated ALT and GGT levels were associated with a higher risk of SRLI, aligning with our results. Our study showed that ALT (corrected *OR* = 1.001 95% confidence interval: 1.001–1.002; *p* < 0.001) and ALT and GGT levels in septic patients with liver damage were significantly greater than those in septic patients without liver damage. GGT is a recognized biomarker of hepatobiliary disease, and logistic regression revealed that GGT (corrected *OR* = 1.001, 95% confidence interval: 1.000–1.002; *p* < 0.001) was a biomarker of hepatobiliary disease. ALT and GGT are primarily found in hepatocytes, and their release into the bloodstream occurs upon cellular damage (20). In sepsis, inflammatory factors such as TNF-*α* and IL-1β stimulate hepatocytes, leading to oxidative stress and microcirculatory disorders, which promote the release of ALT and GGT (21). Concurrent elevation of both enzymes signifies simultaneous damage to hepatocytes and the biliary system (22).

The coagulation dysfunction caused by sepsis can exacerbate liver damage (23). Coagulation dysfunction, characterized by reduced AT III and increased FDP, plays a significant role in exacerbating liver damage (24). The liver synthesizes most clotting factors, and abnormalities can lead to microthrombosis and further hepatocyte injury (25). Our study found that lower levels of AT III and higher levels of FDP were significant predictors of SRLI. This is consistent with the findings of Iba et al. (23), who reported that coagulation abnormalities, particularly reduced AT III levels and increased FDP levels, were associated with a higher risk of sepsis-induced acute liver injury. AT III, a crucial natural anticoagulant protein, plays a significant role in sepsis-related liver injury through several mechanisms: (1) inhibition of coagulation factors (thrombin, factor Xa), thereby preventing microthrombosis formation; (2) suppression of inflammatory factor production (IL-6, TNF-*α*); (3) maintenance of vascular endothelial cell integrity; (4) promotion of hepatocyte regeneration (26–29). FDP also exert complex effects in sepsis-related liver injury, primarily through: (1) elevated levels indicating activation of the coagulation-fibrinolysis system; (2) activation of monocytes and neutrophils, leading to increased release of inflammatory factors; (3) direct damage to vascular endothelial cells; (4) induction of oxidative stress, exacerbating hepatocyte injury (30–32). In conclusion, AT III and FDP influence sepsis-related liver injury through multiple pathways, including modulation of coagulation function, inflammatory responses, and endothelial function.

Interestingly, CO_2_-CP and RDW-CV emerged as independent risk factors for SRLI. CO_2_-CP, an indicator of the function of the kidneys in regulating acid–base balance, has not been extensively studied in the context of liver function damage. However, a study by Wang et al. (33) suggested that reduced CO_2_-CP levels were associated with liver function damage in patients with moderate COVID-19. This finding is consistent with our results, indicating that CO_2_-CP may reflect impaired liver function and metabolic dysfunction in sepsis. RDW-CV measures the variability in red blood cell volume, with higher values indicating an uneven distribution, compromised erythropoiesis, and an unstable intraerythrocytic environment (34). Elevated RDW-CV levels are positively associated with inflammatory cytokines, which are often elevated in sepsis, reflecting systemic inflammation and microcirculatory dysfunction (35).

The AST-to-platelet ratio index (APRI), a marker commonly used in chronic liver diseases, also demonstrated strong diagnostic efficacy for SRLI, with an AUC of 0.797 (36). This suggests that APRI could be a valuable tool for early detection of SRLI in clinical settings, particularly due to its simplicity and cost-effectiveness.

The predictive model we developed, based on ALT, GGT, CO_2_-CP, RDW-CV, AT III, and FDP, demonstrated excellent diagnostic performance with an AUC of 0.890. This model can be readily implemented in clinical practice to aid in the early detection and management of SRLI. Early identification of patients at high risk for SRLI allows for timely intervention, which can include targeted therapies to mitigate liver damage and improve overall patient outcomes. For example, the use of anticoagulants to manage coagulation abnormalities and antioxidants to reduce oxidative stress may be beneficial in these patients.

Moreover, the long-term implications of our findings are significant. Early detection and intervention can lead to reduced morbidity and mortality associated with SRLI. This is particularly important given that sepsis-related liver injury is an independent risk factor for MODS and mortality (37). By improving the early diagnosis and management of SRLI, healthcare providers can potentially reduce the burden of sepsis on healthcare systems and improve patient survival rates. Future studies should focus on validating the predictive model in larger and more diverse patient populations and exploring the effectiveness of targeted interventions based on the identified risk factors. There are several limitations to this study. (1) This is a retrospective study, which may cause selection bias for patients as well as treatment and has a relatively small sample size. (2) The lack of dynamic monitoring of meaningful clinical parameters and dynamic assessment may improve the ability to judge the predictive value of relevant clinical parameters. (3) Other unknown confounding variables still need to be adjusted. Furthermore, it is important to note that this research was conducted in a retrospective manner, with data collection spanning a considerable duration. Consequently, the inclusion of certain patients with incomplete clinical data poses a limitation to the study, ultimately impacting the reliability and validity of the experimental findings.

## 5 Conclusion

Our study combined the indicators suggested to be independent risk factors for SRLI (ALT, GGT, CO_2_-CP, RDW, AT III, and FDP) in a binary logistic regression analysis to predict the occurrence of SRLI.

## Data Availability

The raw data supporting the conclusions of this article will be made available by the authors, without undue reservation.
