# Supplementary material for: Analysis of risk factors for sepsis-related liver injury and construction of a prediction model
Source: Front Public Health. 2024 Dec 6;12:1475292. doi: 10.3389/fpubh.2024.1475292 (PMC11659255; doi:10.3389/fpubh.2024.1475292)
Supplement: Supplementary file 1 [file Table_1.DOCX]

**TABLE S1** Results of the variables VIF > 10 in multicollinearity diagnosis.

| Variables | VIF |
| --- | --- |
| AST | 13.272 |
| LDH | 42.666 |
| DBIL | 1338.269 |
| TP | 181.823 |
| IBIL | 66.13 |
| GLB | 102.72 |
| ALB | 78.764 |
| TBIL | 1671.697 |
| HBDH | 22.086 |
| RBC | 30.643 |
| HGB% | 28.862 |
| MCV | 135.964 |
| MCH | 268.986 |
| MCHC | 168.482 |
| INR | 90.757 |
| PT | 91.109 |

AST, aspartate aminotransferase; LDH, Lactate dehydrogenase; DBIL, direct bilirubin; IBIL, indirect bilirubin; GLB, globulin; ALB, albumin; TBIL, total bilirubin; HBDH, hydroxybutyrate dehydrogenase; RBC, red blood cell; HGB, hemoglobin; MCV, mean cell volume; MCH, mean cell hemoglobin; MCHC, mean cell hemoglobin concentration; INR, international normalized ratio; PT, prothrombin time
