# Supplementary material for: Analysis of risk factors for sepsis-related liver injury and construction of a prediction model
Source: Front Public Health. 2024 Dec 6;12:1475292. doi: 10.3389/fpubh.2024.1475292 (PMC11659255; doi:10.3389/fpubh.2024.1475292)
Supplement: Supplementary file 2 [file Table_2.DOCX]

**TABLE S2 Characteristics of the study cohorts with No Statistically Significant Differences**

|  | Total  n = 1573 | sepsis  n = 1460 | SRLI  n = 113 | *P* value |
| --- | --- | --- | --- | --- |
| CRP(mg/L) | 89.8(32.15-161） | 88.35(31.33-160.75) | 103(46.6-165.5) | 0.244 |
| GLU(mmol/L) | 7.29(5.58-9.63) | 7.34(5.6-9.73) | 6.78(5.15-9.09) | 0.055 |
| NA(mmol/L) | 136.8(132.9-141.1） | 136.8(132.9-141.1) | 136.8(132.65-141.85) | 0.922 |
| CL(mmol/L) | 101.35(97-106.63） | 101.3(97-106.4) | 102.4(96.25-108.65) | 0.269 |
| MG(mmol/L) | 0.79(0.68-0.89) | 0.79(0.69-0.89) | 0.74(0.63-0.93) | 0.081 |
| RBC(10^12^/L) | 3.61(2.99-4.21) | 3.61(3-4.21) | 3.58(2.95-4.29) | 0.973 |
| HGB(g/L) | 106(88-125) | 105(87-125) | 108(90-129.5) | 0.243 |
| MCV(fL) | 90.25(85.8-94.7) | 90.2(85.8-94.6) | 90.3(85.7-97.55) | 0.159 |
| LYMPH(10^9^/L) | 0.87(0.55-1.38) | 0.88(0.55-1.38) | 0.83(0.52-1.32) | 0.186 |
| MONO(10^9^/L) | 0.41(0.23-0.69) | 0.41(0.24-0.69) | 0.47(0.23-0.83) | 0.326 |
| EO(10^9^/L) | 0.03(0.01-0.1) | 0.03(0.01-0.1) | 0.03(0-0.08) | 0.248 |
| BASO(10^9^/L) | 0.01(0-0.1) | 0.01(0-0.02) | 0.02(0-0.04) | 0.090 |

CRP, C-reactive protein; GLU, Glucose; NA, Sodium; CL, Chlorine; MG, magnesium; RBC, red blood cell; HGB, hemoglobin; MCV, mean cell volume; MCH, mean cell hemoglobin; LYMPH, lymphocytes; MONO, monocyte; EO, eosinophil; BASO, basophil;
